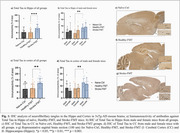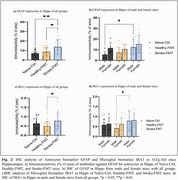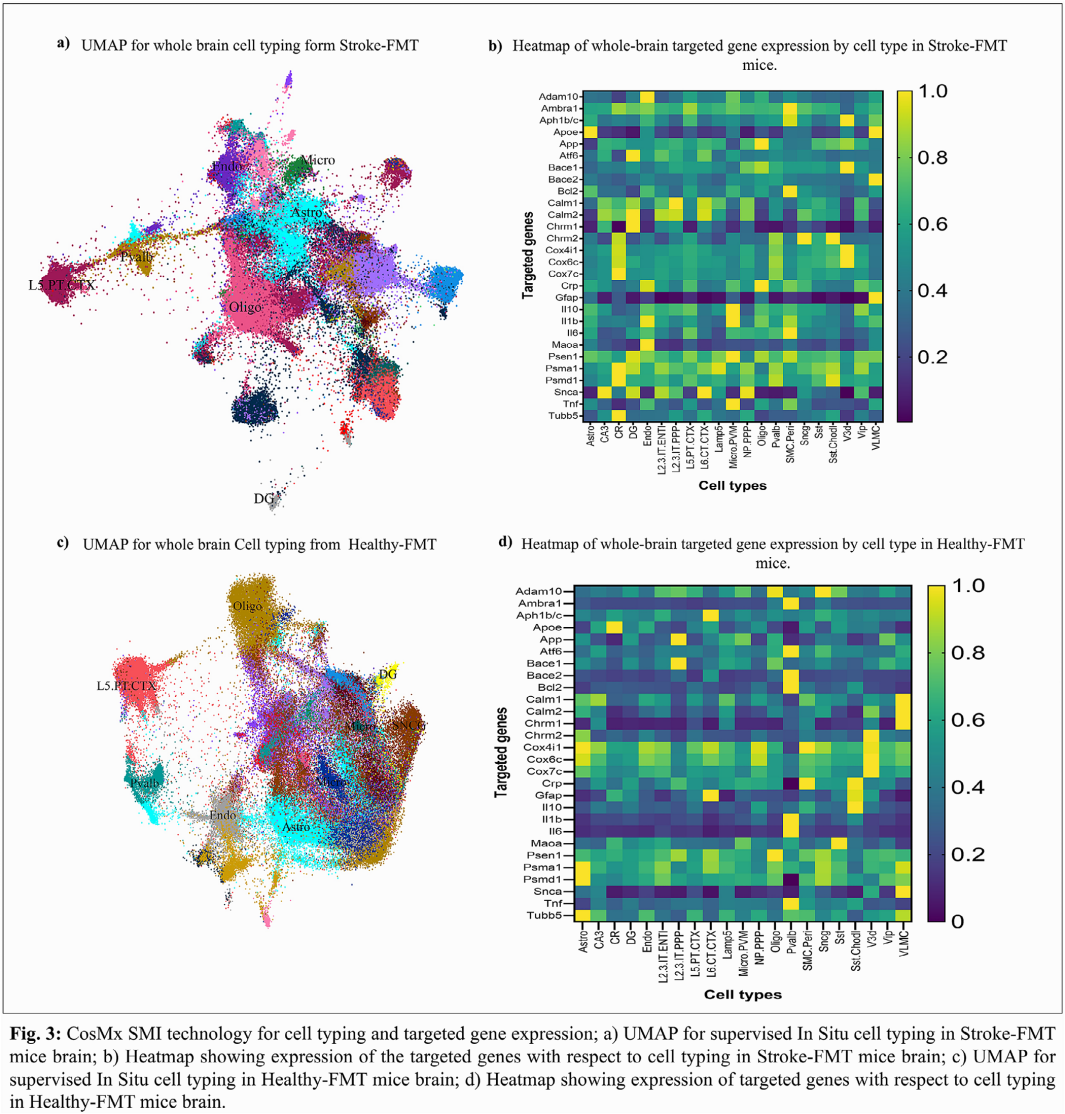# Stroke‐Induced Gut Dysbiosis Increases Alzheimer's Disease Risk: Insights from Immunohistochemistry and Single‐Cell Spatial Transcriptomics

**DOI:** 10.1002/alz70855_106078

**Published:** 2025-12-24

**Authors:** Chetan Aware, Maalavika Govindarajan, Kira Ivanich, Aaron Ericsson, Zezong Gu, Jiankun Cui, Amitai Zuckerman, Lixin Ma, Ai‐Ling Lin

**Affiliations:** ^1^ University of Missouri, Columbia, MO, USA

## Abstract

**Background:**

Stroke increases the risk of Alzheimer's disease (AD), but underlying mechanisms remain unclear. This study investigates whether gut dysbiosis (imbalance in gut microbes) from acute ischemic stroke worsens AD pathology. Using fecal microbiota transplantation (FMT) from stroke patients into 3xTg‐AD mice, we examine its impact on neuroinflammation and AD markers (Total Tau, GFAP, and IBA1). By integrating immunohistochemistry (IHC) and single‐cell spatial transcriptomics, we assess dysbiosis‐driven changes in neuroinflammation, AD pathology, cell typing, and targeted gene expression in the brain to elucidate the gut‐brain axis and explore therapeutic strategies.

**Method:**

Stool samples from stroke patients (*n* = 8) and age‐matched healthy controls (*n* = 8, aged 55‐80 years) were used for FMT in three‐month‐old 3xTg‐AD mice. Mice were randomized into naïve control (male: *n* = 5, female: *n* = 8), Healthy‐FMT (male: *n* = 12, female: *n* = 15), and Stroke‐FMT (male: *n* = 14, female: *n* = 17) groups. FMT followed a one‐week antibiotic treatment. IHC assessed Total Tau (hippocampus and cortex), GFAP, and IBA1 (hippocampus) and CosMx Spatial Molecular Imaging (SMI) analyzed cell type‐specific changes and targeted gene expression in the whole brain of mice.

**Result:**

IHC analysis revealed a significant increase in neuroinflammation and AD pathology in Stroke‐FMT mice, with Total Tau levels significantly elevated in the hippocampus and cortex (*p* <0.001) (Figure 1a, c), and more pronounced increase in males (*p* <0.01) (Figure 1b, d), also shown the representative IHC images for all groups (Figure 1e–g). GFAP expression in astrocytes (Figure 2a, b) and IBA1 expression in microglia (Figure 2c, d) were significantly higher in Stroke‐FMT than Healthy‐FMT (*p* <0.05) in hippocampus, indicating glial activation and neuroinflammation. CosMx‐SMI cell typing showed increased astrocyte and microglia density in Stroke‐FMT, consistent with gut dysbiosis‐induced neuroinflammation compared to Healthy‐FMT in UMAP clustering (Figure 3a, c). Stroke‐FMT mice exhibited dysregulated expressions of ApoE, GFAP, APP, PSEN1, BIN1, and SORL1 across astrocytes, microglia, CA3, and DG, suggesting intensified neuroinflammation and synaptic impairment compared to Healthy‐FMT, potentially exacerbating AD pathology (Figure 3b, d).

**Conclusion:**

Stroke donor FMT‐induced gut dysbiosis exacerbates AD pathology, emphasizing the critical role of the gut‐brain axis in linking stroke and AD. Targeting gut dysbiosis may offer a novel therapeutic strategy for stroke‐related AD progression.